# Alcohol Impairs N100 Response to Dorsolateral Prefrontal Cortex Stimulation

**DOI:** 10.1038/s41598-018-21457-z

**Published:** 2018-02-21

**Authors:** Genane Loheswaran, Mera S. Barr, Reza Zomorrodi, Tarek K. Rajji, Daniel M. Blumberger, Bernard Le Foll, Zafiris J. Daskalakis

**Affiliations:** 1 Translational Addiction Research Laboratory, Centre for Addiction and Mental Health, Toronto, Ontario Canada; 2 Temerty Centre for Therapeutic Brain Intervention, Centre for Addiction and Mental Health, Toronto, Ontario Canada; 3 Biobehavioural Addictions and Concurrent Disorders Laboratory (BACDRL), Schizophrenia Program, Centre for Addiction and Mental Health, Toronto, Ontario Canada; 4 Department of Psychiatry, University of Toronto, Toronto, Ontario Canada

## Abstract

Alcohol is thought to exert its effect by acting on gamma-aminobutyric (GABA) inhibitory neurotransmission. The N100, the negative peak on electroencephalography (EEG) that occurs approximately 100 ms following the transcranial magnetic stimulation (TMS) pulse, is believed to represent GABA_B_ receptor mediated neurotransmission. However, no studies have examined the effect of alcohol on the N100 response to TMS stimulation of the dorsolateral prefrontal cortex (DLPFC). In the present study, we aimed to explore the effect of alcohol on the DLPFC TMS-evoked N100 response. The study was a within-subject cross-over design study. Fifteen healthy alcohol drinkers were administered TMS to the DLPFC before (PreBev) and after consumption (PostBev) of an alcohol or placebo beverage. The amplitude of the N100 before and after beverage was compared for both the alcohol and placebo beverage. Alcohol produced a significant decrease in N100 amplitude (t = 4.316, df = 14, p = 0.001). The placebo beverage had no effect on the N100 amplitude (t = −1.856, df = 14, p = 0.085). Acute alcohol consumption produces a decrease in N100 amplitude to TMS stimulation of the DLPFC, suggesting a decrease in GABA_B_ receptor mediated neurotransmission. Findings suggest that the N100 may represent a marker of alcohol’s effects on inhibitory neurotransmission.

## Introduction

Alcohol is a widely used drug which acts on multiple neurotransmitter systems in the brain^1^. The neurophysiological and behavioural effects of alcohol vary with blood alcohol concentration (BAC). Alcohol is thought to exert its effects by acting on excitatory and inhibitory neurotransmission in the brain. Findings from number of *in vitro* and animal studies suggest that alcohol produces an increase in GABA_A_ receptor mediated neurotransmission^2–5^ and a decrease in glutamatergic neurotransmission^6–9^. However, alcohol’s effects GABA_B_ receptor mediated neurotransmission are less clearly understood.

Transcranial magnetic stimulation (TMS) is a non-invasive experimental technique that can be used to study the effects of various drugs on excitatory and inhibitory neurotransmission in the brain^10,11^. Using TMS with electromyography (EMG), Ziemann *et al*., demonstrated that acute alcohol consumption results in an increase in short-interval intracortical inhibition (SICI) and a prolongation of the cortical silent period (CSP), indices of GABA_A_ and GABA_B_ receptor mediated neurotransmission respectively in the motor cortex^12^. A decreased intracortical facilitation (ICF), an index of NMDA receptor mediated neurotransmission, was also observed. These findings provided evidence for an impairment of cortical excitability and inhibition by alcohol in the motor cortex.

More recently, the combination of TMS with electroencephalography (EEG) has allowed for the measurement of cortical excitability and inhibition directly from the cortex^13,14^ with high temporal precision. The negative peak that occurs ~100 ms following the TMS pulse is the largest TMS-evoked EEG potential^15,16^. This component is believed to represent inhibitory processes, particularly GABA_B_ receptor mediated inhibition^17–19^. Motor measures of long-interval cortical inhibition (LICI) and the CSP, thought to represent GABA_B_ receptor mediated neurotransmission, are correlated with motor TMS-evoked N100 amplitude^17,20^. Similarly, administration of baclofen, a GABA_B_ agonist increases the amplitude of the motor TMS-evoked N100^21^. Together, these findings provide strong evidence that the N100 represents GABA_B_ receptor activities. A study reported that alcohol consumption abolished the N100 response to TMS applied to the motor cortex^22^. The authors speculated that this was due to alcohol altering the cortico-cortical connectivity of the motor cortex and/or an overall suppression of alcohol on the motor cortex.

The impairing effects of alcohol are likely mediated, at least in part, by alcohol’s impairing effect on neurotransmission in the dorsolateral prefrontal cortex (DLPFC), a brain region that plays a key role in brain reward system and cognitive functioning^23^. Using TMS with EEG, Kahkonen *et al*., 2003 reported that consumption of alcohol at a dose of 0.8 g/kg produced a decrease in global mean field amplitude (GMFA) in response to single pulse TMS to the left prefrontal cortex, suggesting reduced excitability in the prefrontal cortex^24^. The current study differs from Kahkonen *et al*. for the following reasons: 1) the present study specifically targeted the DLPFC rather than the left prefrontal cortex 2) the present study evaluated the N100 time window (±20 ms) while Kahkonen *et al*. evaluated a broad time window of 30–130 ms. In the current study, we aimed to evaluate the effect of alcohol on the amplitude of the N100 response to TMS stimulation of the DLPFC.

## Methods

### Study Design

The study was a within-subject, randomized cross-over design. Subjects who met eligibility criteria were enrolled into the study. Following enrollment, subjects attended two study visits. Subjects were required to drink an alcohol beverage during one study visit and a placebo beverage during the other visit. TMS (100 pulses) was administered to the left DLPFC during study visits before (PreBev) and after beverage (PostBev) consumption and EEG was collected. There was a one-month washout period during both study visits and the order of the study visits was randomized.

### Study Visits

Upon arrival of the subject to each study visit, breath measures of carbon monoxide (CO) and BAC were obtained. Urine drug tests were administered to all subjects prior to each study visit and urine pregnancy tests were administered to all females of child-bearing age. The study visit was only commenced if urine drug tests, urine pregnancy tests and BAC measures were negative. The resting motor threshold (RMT) and the stimulus intensity required to produce an average motor evoked potential (MEP) amplitude of 1 mV (1 mV_T1_) was obtained. The DLPFC was identified using the F5 electrode as the marker^25^. Assessment of baseline TEPs was performed by applying a train of 100 pulses at 0.1 Hz at stimulus intensity 1 mVT_1_ (PreBev). In order to achieve a rapid increase in BAC, subjects were given 15 minutes to consume the beverage. BAC was obtained via breath measures every 15 minutes after beverage consumption. The stimulus intensity necessary to produce an average MEP of 1 mV (1 mV_T2_) was reassessed when each subjects BAC reached ≥17.4 mM (≥0.08%). Another train of 100 pulses at 0.1 H was administered to the left DLPFC at stimulus intensity 1 mVT_2_ (PostBev). EEG was collected throughout both testing sessions.

### Subjects

Subject recruitment was done through ads posted at the University of Toronto as well as online ads (Craigslist, Kijiji, etc). Informed consent was provided by all subjects prior to participation in the study and all experiments were conducted in accordance with the Declaration of Helsinki. The study was approved by the research ethics board at the Centre for Addiction and Mental Health. Fifteen healthy alcohol drinkers (mean age 33.42, ±7.52, 23–46 years of age, 10 Males) who had endorsed at least one heavy drinking episode (defined at 5 standard drinks for men and 4 standard drinks for women within two hours) (“NIAAA Guidelines”, 2004), within the last month, as assessed using the Alcohol Timeline Follow-Back^26^, participated in the study. Subjects were non-smokers (had not smoked any cigarettes in the last three months) and were between the age of 19 to 60 years of age. Subjects did not meet did not meet DSM-IV criteria for any current drug abuse or dependence or any psychiatric disorders. Subjects were excluded if they had a history of seizures, neurological disease or cognitive impairment (determined by a score of <24 on the Mini Mental State Examination)^27^. None of the subjects reported regular use of any therapeutic or recreational psychoactive drugs during the last three months.

### Beverages

Beverages consisted of either an alcohol beverage made with 95% United States Pharmacopia (USP) alcohol or a placebo beverage of equal volume made of orange juice and tonic water. The alcohol beverage was mixed in a 1:5 ratio with orange juice and tonic water. Both the placebo and alcohol beverages had 0.2 mL of Absolute Vodka (0.2 mL of 40% alcohol) added on top of the beverage immediately before beverage administration to the subjects. This very small amount of alcohol was added to the beverage to produce the odor of alcohol without producing any of alcohol’s effects. Subjects were randomized to receive either alcohol or placebo beverage first and received the other beverage in the subsequent visit. Subjects were blinded to the type of beverage they received. Investigators were also blinded to the type of beverage until after the first BAC measure was obtained 15 minutes following beverage consumption.

### BAC and CO Measurements

An Alco-Sensor FST (DAVTECH Analytic Services, Canada) was used to obtain a breath sample at the beginning of all study visits and at 15 min intervals following beverage consumption. CO measures were obtained at the beginning of all study visits using Micro+™ Smokerlyzer® CO monitor (Bedfont Scientific Ltd.)

### TMS Stimulation

The RMT and 1 mV intensity were obtained by applying TMS pulses over the left motor cortex using a 7 cm figure-of-eight coil and two Magstim 200 stimulators (Magstim Company Ltd, UK) connected via a Bistim module. The site of stimulation in the left motor cortex was determined by identifying the optimal position for eliciting MEPs from the right abductor pollicis brevis (APB) muscle. Two disposable disc electrodes over the right APB muscle in a tendon-belly arrangement were used to capture EMG. The EMG signal was amplified using a Model 2024 F amplifier (Intronix Technologies Corporation, Bolton, Ontario Canada) and filtered at band pass of 2 Hz to 5 kHz and digitized using the Micro 1401 (Cambridge Electronics Design, Cambridge UK). RMT was evaluated using the protocol outlined by Rossini *et al*.^28^. The RMT was defined as the minimum stimulus intensity that elicits a MEP of more than 50 µV in five of ten trials. The intensity of stimulation was determined based on the intensity required to produce a mean peak-to-peak MEP amplitude on 1 mV, which corresponded to approximately 120% of the RMT. Dedicated software (Cambridge Electronic Design, UK) was used to collect electromyography data. Once the 1 mv intensity was determined, TMS pulses were applied to the left DLPFC for the test paradigms. TMS stimuli were administered over the F5 electrode at 1 mV intensity at 0.1 Hz during all test paradigms.

### EEG Data Collection and Analysis

A 64-channel Synamps 2 EEG system was used to acquire EEG data. The electrode positioned posterior to the Cz electrode was used as the reference electrode. The impedance of each of the electrodes (Ag/AgCl ring electrodes) was lowered to <5 kΩ. To monitor eye movement artefacts, four electrodes were placed on the outer corner of each eye, as well as, above and below the left eye, to monitor the eye movement artefact. In order to minimize TMS related artefacts and avoid saturation of the amplifiers, EEG signals were recorded using DC and a low pass filter of 200 Hz at 20 kHz sampling rate^14^.

EEG data was down-sampled to 1000 Hz. The data was segmented was −1000 ms to 2000 ms relative to the TMS pulse. Baseline correction was performed with respect to the pre-stimulus interval. To eliminate EEG artifacts, the EEG data was re-segmented from 25 ms to 2000 ms. The data was then digitally filtered using a second order Butterworth zero-phase shift 1–55 Hz band pass filter (24 dB/Oct). The recordings from both sessions (PreBev, PostBev) were concatenated to apply the same criteria to both recordings to remove noise from the data.

Initially, EEG data were visually inspected to eliminate trials and channels that were highly contaminated with noise (muscle activity, electrode artifacts). Then, an electrodes-by-trials matrix of ones was created and assigned a value of zero if an epoch had: (1) an amplitude larger than +/− 150 μV; (2) a power spectrum that violated the 1/f power law; or (3) a standard deviation 3 times larger than the average of all trials. Additionally, electrodes were rejected if their corresponding row had more than 60% of columns (trials) coded as zeros and epochs were removed if their corresponding column had more than 20% of rows (electrodes) coded as zeros. Lastly, an independent component analysis (ICA) (EEGLAB toolbox; Infomax algorithm) was performed to remove eyeblink traces, muscle artifacts, and other noise from the EEG data and data was re-referenced to the average for further analysis.

TMS evoked potential (TEP) was calculated by averaging the response over all epochs for each of the sessions. The Hilbert transformation was then used to determine the area under the instantaneous amplitude between 50–275 ms after TMS onset. The first interval (i.e., 50 ms) was chosen because it represents the earliest artifact-free data and the second interval (i.e., 275 ms) was chosen to cover the activity of GABA_B_ receptors^29,30^.

### N100 Analysis

The amplitude of the N100 was calculated by measuring the average amplitude under the curve of the global mean field amplitude (GMFA) at 100ms ± 20ms. The GMFA is used to index global field activity and is measured by calculating the root mean squared value of the CEA across electrodes^31^. The GMFA was used to calculate in order to capture the N100 regardless of which electrodes it may occur over.

## Results

### 1 mV Intensity (% stimulator output)

Paired t-tests were conducted to examine if the alcohol or placebo beverage had an effect on 1 mV peak-to-peak intensity between PreBev and PostBev. There was no significant difference between 1 mV_T1_ and 1 mV_T2_ for placebo (t = −1.740; df = 14; *p* = 0.104) or alcohol (t = −0.893; df = 14; *p* = 0.387) beverage, suggesting that neither alcohol nor placebo beverage had an effect on corticospinal excitability (Table 1).

**Table 1:** TMS Parameters.

	Alcohol T1	Alcohol T2	Placebo T1	Placebo T2
Resting motor threshold (% stimulator output)	58 ± 8	—	59 ± 8	—
1 mV Intensity (% stimulator output)	71 ± 12	72 ± 14	71 ± 11	71 ± 11

^*^Values are in Mean ± 1 Standard Deviation (SD).

### Breath Alcohol Concentration

The mean peak BAC was 23.6 mM ± 4.1 mM (range 18.5 mM-34.2 mM). BAC was always at 0 mM at PreBev and peaked during PostBev. BAC remained above 17.4 mM (the legal intoxication level) throughout the study.

### Effect of Alcohol on N100 Amplitude

**Figure 1:**
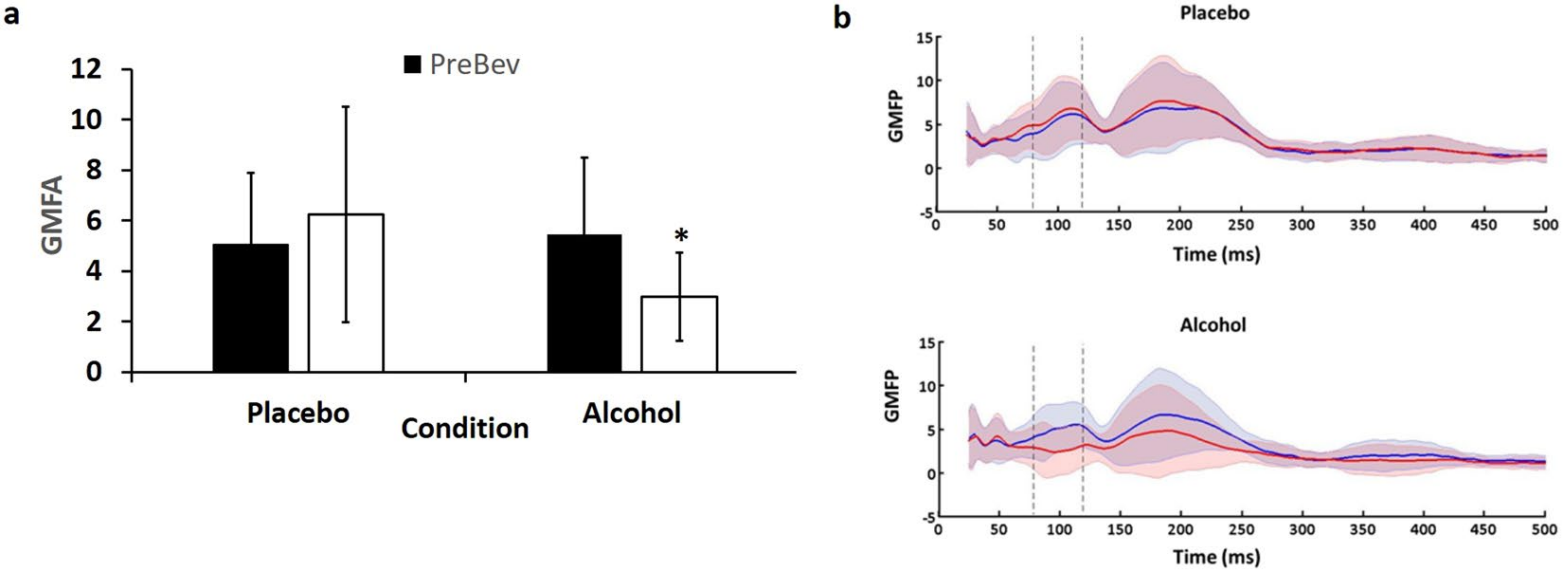
N100 Amplitude. (**a**) Average global mean field amplitude (GMFA) of 100 TMS pulses to the DLPFC before (PreBev) and after (PostBev) the placebo and alcohol beverages (n = 15). Error bars represent the standard deviations. Alcohol significantly reduced the mean PAS-induced neuroplasticity. (**b**) Average global mean field potential before (solid blue line) and after (solid red line) for placebo and alcohol beverages. The standard deviations are marked with the corresponding faint blue and red regions.

The repeated measures ANOVA revealed a beverage by time interaction (F = 26.60; df = 1,14; p < 0.001). Post hoc analyses revealed that alcohol produced a significant decrease in N100 amplitude indicating reduced N100 amplitude at PostBev compared to PreBev (t = 4.316, df = 14, p = 0.001; Fig. 1). There was no significant time effect with the placebo beverage (t = −1.856, df = 14, p = 0.085; Fig. 1).

## Discussion

The current study was aimed at examined the effect of acute alcohol consumption on N100 amplitude, a cortical marker of GABA_B_ receptor mediated neurotransmission. The study found that acute alcohol consumption produces a significant decrease in the N100 amplitude compared to the placebo beverage. These findings suggest that acute alcohol consumption significantly disrupts GABA_B_ receptor mediated neurotransmission.

### Effect of Alcohol on N100 Response to TMS Stimulation of DLPFC

Findings from the current study demonstrated that alcohol produces a significant decrease in the N100 response to DLPFC TMS stimulation. These findings are in accordance with a previous study that examined the effect of alcohol consumption (at a dose of 0.8 kg/kg) on motor cortex TMS stimulation induced N100 response^22^. The authors found that the N100 amplitude was reduced in ten electrodes with the most pronounced N100 component (based on visual assessment) following alcohol consumption. Our current findings demonstrate that alcohol also decreases the N100 amplitude in response to TMS of the DLPFC. These findings suggest that alcohol has a suppressive effect on the DLPFC similar to that previously observed in the motor cortex, suggesting widespread effects of alcohol.

The reduction of N100 amplitude by alcohol, suggestive of a decrease in GABA_B_ receptor neurotransmission, may be difficult to reconcile at first with the commonly accepted notion that alcohol produces an increase in GABAergic neurotransmission^2–5^. However, while alcohol has been demonstrated to result in an increase in GABA_A_ receptor mediated neurotransmission^2–5^, its effect on GABA_B_ receptor mediated neurotransmission is less well understood. Similar to the present study, Kahkonen & Wilenius (2007) reported a decrease in motor TMS-evoked N100 amplitude following acute alcohol consumption^22^. Additionally, administration of GABA_A_ agonists such as alprazolam and diazepam produce a decrease in N100 amplitude in response to TMS stimulation to the motor cortex, similar to alcohol^32^. It has been argued that the decrease in N100 amplitude produced by GABA_A_ agonists may be due to inhibition of GABA_B_ receptor mediated inhibitory post-synaptic potentials in the neocortex and hippocampal pyramidal neurons by GABA_A_ receptor activation^32–34^. Given that alcohol is also agonistic at the GABA_A_ receptor, a similar mechanism may underlie the reduction in N100 amplitude by alcohol. Alternatively, alcohol’s antagonistic effect at NMDA receptors may contribute to a loss of activation of GABAergic neurons by alcohol and produce a reduction in GABA_B_ receptor mediated neurotransmission^35^.

### Implications for Alcohol Use Disorders and Treatment

The reduction in N100 amplitude caused by alcohol suggests that the N100 may also be affected following chronic alcohol abuse and in individuals who are alcohol dependent. Indeed, previous studies have reported a decrease in N100 amplitude during laboratory cognitive tasks in alcohol dependent individuals compared to healthy controls^36,37^. Given these findings, the N100 may serve as a useful marker of alcohol use disorders. Future studies can confirm whether a similar impairment is observed in TMS-evoked N100 response in patients with alcohol use disorders and whether treatment for alcohol use disorders is associated with regulation of the N100 response.

The following study has a number of limitations. Firstly, it would have been useful to include other TMS measures of cortical inhibition, such as LICI, to confirm alcohol’s impairing effect of GABA_B_ receptor mediated transmission. However, given that previous studies have confirmed that LICI is correlated to N100 amplitude, we can infer a similar impairing effect would be observed on this measure. Another limitation of the study is that BAC was calculated from breath rather than blood samples. However, breath measures have been demonstrated to correlate highly with blood measures and prevent unnecessary discomfort to the subjects. Lastly, the sample size of the current study was relatively small. However, given the within-subjects design and the large effect of alcohol on N100 amplitude, the sample size was sufficient for the purpose of this study.

## Conclusions

In conclusion, findings from the current study demonstrate that alcohol causes a significant reduction in N100 amplitude in response to TMS stimulation of the DLPFC, suggesting that alcohol results in a reduction of GABA_B_ receptor mediated neurotransmission. Findings from the current study suggest that the N100 can act as marker of alcohol’s effects on inhibitory neurotransmission. Future studies may explore whether the TMS-evoked N100 is affected in alcohol dependent individuals and whether the N100 is normalized following treatment for alcohol use disorders.
